# Leukemia stem cell-bone marrow microenvironment interplay in acute myeloid leukemia development

**DOI:** 10.1186/s40164-021-00233-2

**Published:** 2021-07-10

**Authors:** Yiyi Yao, Fenglin Li, Jiansong Huang, Jie Jin, Huafeng Wang

**Affiliations:** 1 Department of Hematology, The First Affiliated Hospital, College of Medicine, Zhejiang University, 79 Qingchun Road, Hangzhou, 310003 Zhejiang People’s Republic of China; 2 Zhejiang Provincial Key Lab of Hematopoietic Malignancy, Zhejiang University, Hangzhou, 310003 Zhejiang People’s Republic of China; 3 Zhejiang Laboratory for Systems & Precision Medicine, Zhejiang University Medical Center, Hangzhou, 310000 Zhejiang People’s Republic of China

**Keywords:** Acute myeloid leukemia, Bone marrow microenvironment, Leukemia stem cell, Interaction, Environment-mediated drug resistance

## Abstract

Despite the advances in intensive chemotherapy regimens and targeted therapies, overall survival (OS) of acute myeloid leukemia (AML) remains unfavorable due to inevitable chemotherapy resistance and high relapse rate, which mainly caused by the persistence existence of leukemia stem cells (LSCs). Bone marrow microenvironment (BMM), the home of hematopoiesis, has been considered to play a crucial role in both hematopoiesis and leukemogenesis. When interrupted by the AML cells, a malignant BMM formed and thus provided a refuge for LSCs and protecting them from the cytotoxic effects of chemotherapy. In this review, we summarized the alterations in the bidirectional interplay between hematopoietic cells and BMM in the normal/AML hematopoietic environment, and pointed out the key role of these alterations in pathogenesis and chemotherapy resistance of AML. Finally, we focused on the current potential BMM-targeted strategies together with future prospects and challenges. Accordingly, while further research is necessary to elucidate the underlying mechanisms behind LSC–BMM interaction, targeting the interaction is perceived as a potential therapeutic strategy to eradicate LSCs and ultimately improve the outcome of AML.

## Introduction

Acute myeloid leukemia (AML) is one of the most common acute leukemia in adults, which is characterized by abnormal proliferation of undifferentiated and nonfunctional hematopoietic blasts in bone marrow accompanied by injured production of normal blood cells [1]. Global incident cases of AML gradually increased from 63,840 in 1990 to 11,957 in 2017 according to latest epidemiological data from Global Burden Disease 2017 [2, 3]. Over the past 40 years, administration of daunorubicin for 3 days then with continuous infusion of cytarabine for 7 days has been the standard induction regimen for AML without encouraging breakthrough. Due to the prominent problems of chemotherapies and targeted therapies including treatment-related toxicity and high therapeutic resistance rates caused by residual leukemia stem cells (LSCs), the efficacy of current treatments for AML has been unsatisfactory. The relapse rate is up to 60% in higher risk AML patients, leading to an overall 5-year survival rate of about 40% in patients under 60. Meanwhile, a relatively grim prognosis was found in elderly patients over 60 years old, with the overall 5-year survival rate declining rapidly to only about 10% [4].

Bone marrow microenvironment (BMM), also known as bone marrow niche, was first depicted by Schofield in 1978 [5]. Schofield et al. proposed that besides structural and supportive roles, the specific microenvironment intimately engaged in maintaining long-term self-renewal of hematopoietic stem cells (HSCs). Later investigations identified distinct components of BMM in murine models and confirmed that the interaction of BMM and HSCs participated in governing the fate of HSCs [6, 7]. Of note, heterogeneous niche in bone marrow differs in composition and thus exerts diverse functions on modulating HSC behaviors [8]. LSCs are a group of leukemic cells that maintain the vitality of leukemic cell populations through aberrant self-renewal capacity and uncontrolled immortal proliferation status. Residual LSCs in the bone marrow microenvironment after chemotherapy is generally regarded as the key factor for AML relapse [9]. Recently rising evidence demonstrated that not only accumulating genetic lesions but also BMM's runaway regulation of HSCs participated in the transition from HSCs into LSCs [10]. The transformed LSCs not only initiated hematopoietic homeostasis breaking, but also promoted remodeling of the surrounding BMM [11]. Further, at the expense of healthy HSCs and their surrounding stromal cells, hijacked BMM accelerated the progression of AML and led to chemo-resistance by providing anti-apoptotic shelter [12]. Taken together, the LSC–BMM interaction plays a crucial role in AML development through a combination of soluble factors, adhesion signals, and neural signals. Thus, there rises an urgent need for a comprehensive understanding of LSC–BMM interactions to expand novel specific targets and develop therapeutic regimens targeting LSC mobilization and elimination. Here, we briefly addressed several BMM components closely associated with hematopoiesis regulation, then concentrated on the interactions between BMM and LSCs in AML development and chemo-resistance, and summarized the potential therapeutic strategies targeting LSC–BMM interplay.

## Constructions of BMM

Abundant blood vessels and nerves systems were contained in BMM. After the branches of nutrient arteries penetrate through cortex via nutrient foramen, they give off widespread small arterioles which are wrapped by innervated smooth muscle cells to permit alteration of vessel caliber and modulation of flow resistance. The small arterioles run closed to the endosteum and extend into the medullary cavity to form a dense network of sinusoids. Sinusoidal network is considered to be the major site permitting efficient exchange of oxygen, nutrients, cytokines and hormones molecules, removing metabolic waste products, as well as providing channels for mature hematopoietic cells to enter peripheral blood. The sinusoidal vessels then influx into the central venous sinus, and ultimately outflow to nutrient veins which pass through nutrient foramen to exit bone marrow and enter the extramedullary blood circulation [7].

Based on anatomical structure, BMM has been generally categorized into two specialized niches: perivascular and endosteal. The perivascular niche (also known as the endothelial niche) has been further divided into perisinusoidal region and periarteriolar region according to the blood vessel types passing through. The endosteal niche (also known as the osteoblastic niche) is located on the endosteal bone surface, where osteoblasts are primarily lined. Perisinusoidal region was reported distant from endosteal niche, while periarteriolar region located adjacent to endosteum surface [13]. The earliest investigations in murine models suggested that the majority of primitive hematopoietic cells located near the endosteal surface rather than central medullary regions [14]. Later, emergence of new evidence has changed previous embedded perspectives. A research using deep confocal imaging reported the opposite discovery that the majority of HSCs located in the central bone marrow area distant away from the endosteum and accumulated in the diaphysis instead of the metaphysis regions of long bone [15]. Moreover, the controversy over whether spatial distribution differences of activated/quiescent HSCs exist has continued. Kunisaki et al. [16] reported that quiescent HSC populations tended to locate in periarteriolar region. However, later another study addressed that the majority of HSC population primarily surrounded around sinusoidal blood vessels instead of arterioles with no significant differences in the spatial location between dormant and proliferating HSC populations [15]. A temporarily accepted view is that while the majority of HSCs is located around sinusoids rather than proximal to arterioles or bone surface, the advantage in abundance does not represent a difference in HSC function and status [17]. However, whether there is a potential specific niche supporting HSC quiescence or activation is still worth considering. Indeed, a latest study identified distinct cavity types in the metaphysis of long bones in mice, implying that the traditional way of HSC niche characterizing as endosteal or perivascular is inadequate [18].

Transgenic murine models have been widely employed to identify the constitution of the diverse and complex BMM. Besides hematopoietic cell populations, the BMM is mainly comprised of heterogeneous stromal cell populations and extracellular matrix (ECM). Currently, stromal cell populations including mesenchymal stem cells (MSCs; also known as mesenchymal stromal cells), arteriolar and sinusoidal type endothelial cells, osteoblasts, osteoclasts, osteocytes, macrophages, megakaryocytes, adipocytes, sympathetic neurons, non-myelinating Schwann cells, C-X-C motif chemokine ligand 12 (CXCL12)-abundant reticular cells, and ECM components like collagen, fibronectin, laminin, plasmin have been identified in BMM [7]. In this section, we focused on normal hematopoietic microenvironment of bone marrow and described several crucial components associated with hematopoiesis.

### MSCs

MSCs, primarily wrapped around arterial and sinusoidal vessels, are a heterogeneous population of non-hematopoietic stem cells. Evidence supported that MSCs displayed the potential to give rise to a wide range of mature mesenchymal cell populations including osteoblasts, chondrocytes, adipocytes and fibroblast-like cells in the stromal microenvironment [19]. Currently, wide interest has been elicited to MSC-derived extracellular vehicles (MSC-EVs). MSC-EVs contain a variety of proteins and RNAs, express both EV and MSC markers, and interact with targeted cells by transporting cargos into the intracellular compartment. Several specific EV-derived miRNAs have been reported to be participated in immunomodulation and regulation of MSC differentiation, proliferation and angiogenic activity [20].

### Bone marrow adiposes

Bone marrow adipose tissue (BMAT) refers to MSCs-derived adipose tissue in the bone marrow, which is an extramedullary reservoir of normal HSCs. Adipose tissue in different compartments perform distinct functions, which set BMAT apart from other adipose tissues like white adipose tissue and brown adipose tissue. There has been consistent controversy about the regulation direction of BMAT in hematopoiesis in the past years [21, 22]. Zhou et al. [23] reported that there existed functional differences with adipocytes in different bone marrow compartments (long bones versus caudal vertebrae) in mice, with adipocytes promoting HSCs maintenance and regeneration by secreting stem cell factor (SCF) in long bones while making suppressive effects on hematopoiesis in caudal vertebrae. Besides, Zhou et al. also proposed a new perspective that adipogenesis was an emergency response to cytopenia that could promote rapid hematopoiesis, which emphasized the strongly connected association between BMAT and hematopoiesis. The regulation of BMAT on hematopoiesis may be largely depend on the dynamic hematopoietic environment itself, with further studies to verify.

### Autonomic nervous system

Evidence supported the positive role of autonomic nervous system to form a circadian rhythm of HSC mobilization through rhythmically CXCL12 down-regulating and physiologic oscillations of glucocorticoid signal [24–28]. Recently, A study found that the central (parasympathetic) and local (sympathetic) cholinergic signals cooperated to regulate the migration of hematopoietic stem/progenitor cells (HSPCs) and leukocytes, thereby generating circadian rhythmic oscillations [29]. Besides participating in mobilization, the autonomic nervous system has been reported to highly engaged in regulating the flow of arterioles and differentiation of osteoblasts [7, 30].

Neuroglial cells have been shown to be involved in size adjustment of the HSCs pool instead of purely conduiting as supportive cells. Indeed, non-myelinating Schwann cells encapsulating autonomic nerves maintained surrounding HSCs dormant by activating latent transforming growth factor β (TGF-β) [31]. Sympathetic nerve denervation decreased TGF-β-producing cell population, resulting in a rapid loss of HSCs from bone marrow [32].

### Extracellular matrix

The ECM network, abundant with collagens, glycoproteins, and proteoglycans, functions in compartmentalization of bone marrow and participates in cell adhesion, migration, and proliferation [33, 34]. Several ECM ingredients in hematopoiesis have been investigated. For instance, an early study has shown that α5-containing laminin favored multipotent hematopoietic cell adhesion [35]. Bone marrow from laminin α4 deficient mice promoted the quiescence of HSCs and impaired the recirculation of HSCs across bone marrow vessels into the bone marrow niche [36]. Osteopontin (OPN), a phosphor glycoprotein which mainly secreted by osteoblastic cells, was found to induce HSCs migration toward the endosteal region and negatively adjustment pool size and function of HSCs by suppressing the expression of Jagged1 and Ang-1 [34, 37].

### Other components in the endosteal niche and the perivascular niche

It has been reported that osteoblasts within the endosteal niche play a vital role in HSCs long-term maintenance and bone marrow retention [7]. Osteoblasts inhibit the differentiation and enhance the self-renewal of HSCs to maintain HSCs pool through signal pathways including Jagged-1/Notch, Ang-1/Tie-2 and TPO/MPL [38–40]. Visnjic et al. [41] showed that ablation of osteoblasts led to the loss of bone marrow cellularity, followed by a decrease in bone marrow hematopoiesis, accompanied with an active extramedullary hematopoiesis. Moreover, osteoblasts secrete varied hematopoietic cytokines and extracellular proteins, including granulocyte colony-stimulating factor (G-CSF), interleukins, type I collagen, osteocalcin (OCN), OPN and others [42]. Accordingly, these studies have displayed the instrumental role of endosteal niche in HSCs maintenance and HSCs pool regulation.

The perivascular niche is composed of endothelial cells and a range of heterogeneous mesenchymal cells, like CXCL12-abundant reticular (CAR) cells, nestin-GFP^+^ cells, and leptin receptor (LepR)^+^ cells. Evidence showed that these stromal cell subgroups displayed a high degree of overlap with each other in the bone marrow [43]. Adipogenic and osteogenic capacities of CAR cells are regulated by complex transcription factor network to permit enough space reserved for hematopoiesis [44, 45]. CAR cells also secrete chemokine CXCL12 and a series of adhesion molecules to mediate HSCs homing and support HSCs maintenance [46–48]. The distribution of nesting-GFP^+^ cells has been reported associated with GFP expression levels, with nesting-GFP^bright^ mainly distributed around arterioles, while nesting-GFP^dim^ proximal to sinusoids [16]. Such heterogeneous distribution has been reported associated to the regulation of HSCs quiescence and the maintenance of the HSCs pool, despite of the dispute in respective proliferating/dormant HSCs distribution [16].

### Hypoxia and reactive oxygen species (ROS) levels

It has been clarified that the bone marrow microenvironment is ubiquitously hypoxic, with oxygen tension below 10 mmHg [49]. Contrary to the previous view that the endosteum is relatively hypoxic in BMM, Spencer et al. [13] demonstrated a moderate oxygen tension with the increasing distance away from the endosteum toward perivascular niche. Indeed, they found that despite of oxygen exchange taken place between sinusoids and surrounding tissue, the oxygen level was significantly lower in the perisinusoidal region than periarteriolar region. Hypoxia has been previously considered critical for HSCs maintenance. Nevertheless, a latest study based on live-animal imaging reported no HSCs were detected in the region with the lowest oxygen in central BM, indicating that extreme hypoxia may not be the prerequisite for maintaining stem cell quiescence [18]. Additionally, while it is generally reputed that the adaptation to hypoxia is principally mediated through the heterodimeric transcription factor hypoxia-inducible factor (HIF), several investigations reported no detectable effects on HSCs maintenance in inducible acute deletion of Hif-1α and Hif-2α [50–52].

In addition, increasing evidence supported that ROS played a critical role of intracellular in HSCs behavior and function. It has been reported that knockdown of *HIF1-α* and *HIF2-α* caused damage to the self-renewal ability of HSPCs through enhanced ROS production [53]. Emerging studies have focused on the relationship between ROS levels and HSCs maintenance [54, 55]. Itkin et al. indicated that low ROS levels in the poor permeable periarteriolar region appeared to strengthen the maintenance of HSCs quiescence, whereas the ROS levels in the hyperpermeable perisinusoidal region drove activation of HSCs.

## BMM–LSC interaction in leukemia development

**Fig. 1:**
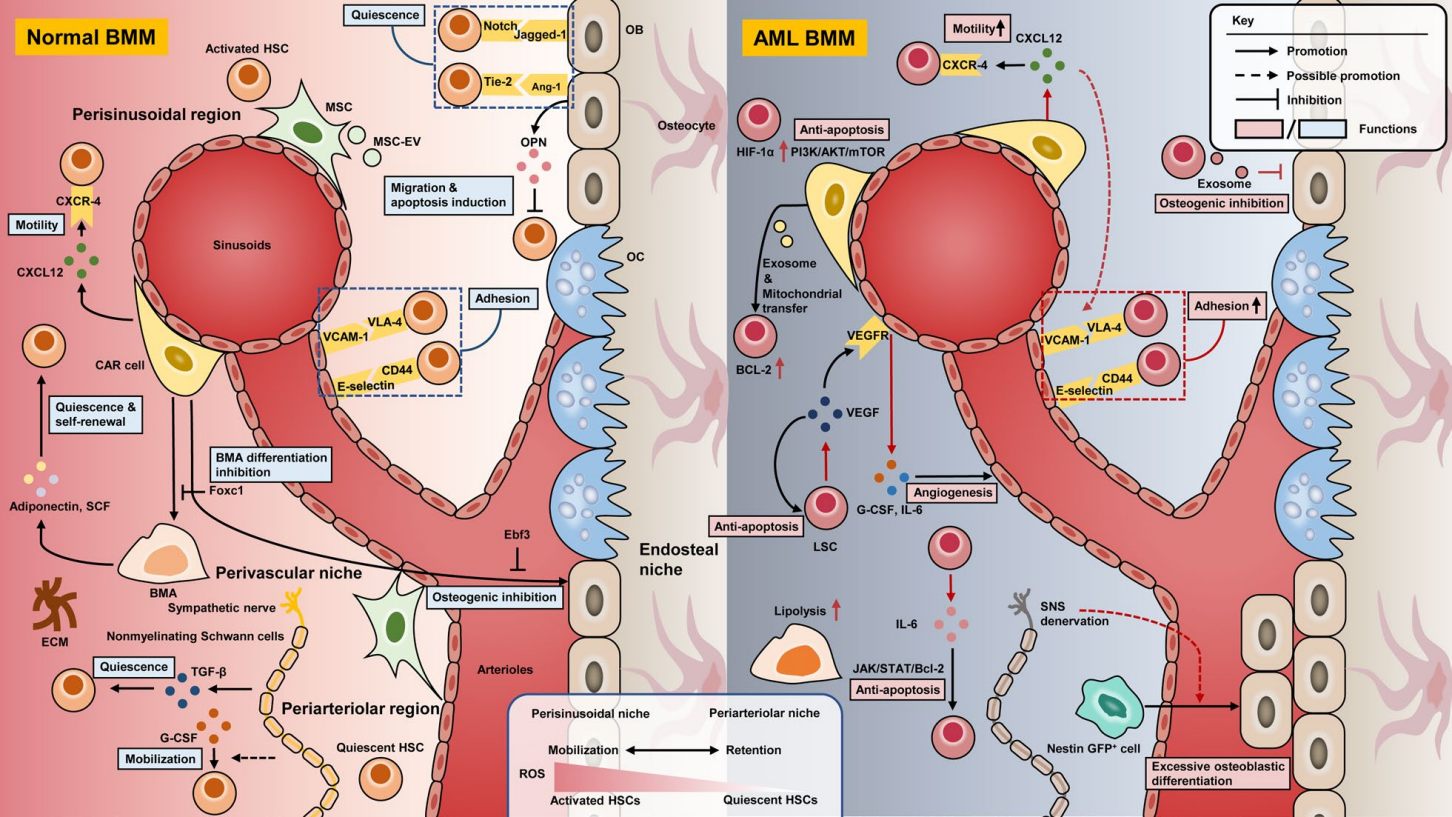
The comparison between normal/AML BMM with associated cellular interactions. The composition of the BMM contains hematopoietic cells, several stromal cell populations as well as ECM. HSCs with different behaviors have been found to reside in heterogenous niches. BMM supports hematopoiesis through interactions mediated by cell–cell contact and soluble secreted factors. Compared to normal BMM, there have been several prominent changes in AML BMM, including differential remodeling of the vasculature, alteration of cytokines secretion together with adhesion capacity, adaptability to hypoxia microenvironment and maintenance of low ROS, which lead to AML development and further chemoresistance. *AML* acute myeloid leukemia, *BMM* bone marrow microenvironment, *HSC* hematopoietic stem cell, *MSC* mesenchymal stem cell, *CXCL12* C-X-C motif chemokine 12, *CXCR4* C-X-C chemokine receptor 4, *VLA-4* very late antigen 4, *VCAM-1* vascular cell adhesion molecule 1, *TGF-β* transforming growth factor-β, *OPN* osteopontin, *G-CSF* granulocyte-colony stimulating factor, *ECM* extracellular matrix, *BMA* bone marrow Adipose, *OB* osteoblast *OC* osteoclast, *Ebf3* transcription factor early B-cell factor 3, *Foxc1* transcription factor forkhead box C1, *HIF* hypoxia-inducible factor, *VEGF* vascular endothelial growth factor, *SNS* sympathetic nervous system, *GFP* green fluorescent protein, *MSC-EV* MSC-derived extracellular vehicles, *ROS* reactive oxygen species, *BCL-2* B-cell lymphoma-2

Accumulating evidence have suggested that AML cells reshape a supportive microenvironment to accelerate leukemia progression and suppress the normal hematopoiesis. Several instrumental molecular players in the regulation of angiogenesis, BMAT remodeling, adhesion factors, neural signals, and hypoxia have been found closely correlated with ‘LSC-educated BMM’ and ‘microenvironment-accelerated AML development and chemo-resistance’. In addition, Kumar et al. [56] revealed that AML-derived exosome secretion played a critical role in the forming of a leukemia growth-permissive and normal hematopoiesis-suppressive niche, which uncovered a novel feature of AML pathogenesis. The comparison of normal BMM and leukemic microenvironment is illustrated in Fig. 1.

### Angiogenesis

Angiogenesis, defined as the development of new blood vessels starting from pre-existing vessels, is distinctly different from the normal vascularity. Driven by the overexpression of pro-angiogenic factors triggered by relative hypoxia in tissues, angiogenesis promotes proliferation of malignant cells by continuous providing oxygen, nutrients, and growth factors from surrounding microenvironment. Bone marrow microvessel density (MVD) can be used to estimate angiogenesis in leukemic patients. Due to the non-solid malignancy feature of AML, initially few studies had emphasized the role of angiogenesis in AML development. However, recent studies on BMM vasculature have found an increasing level of MVD in AML patients and suggested that angiogenesis is highly associated with leukemia progression. Consistent with reports in solid tumors, a poor prognosis was found in high baseline MVD patients [57].

Several angiogenic cytokines and signaling pathways were identified in angiogenesis. Vascular endothelial growth factor (VEGF) and angiopoietin are among the most pivotal angiogenic cytokines secreted by several types of stroma cells and leukemic cells. In general, AML cells secrete VEGF to activate VEGF receptor expressed on both AML cells and endothelial cells [58]. On the one hand, the autocrine form activates BCL2 family to protect leukemic cells from apoptosis [59]. On the other hand, VEGF binding to endothelial cells stimulates growth factors including G-CSF and IL-6 secreted by endothelium, which promote angiogenesis and play critical roles in AML cells survivability and proliferation [60]. Additionally, several studies reported that the up-regulation of VEGF in AML blasts has been closely associated with increased failure of complete remission (CR) and low overall survival (OS) [61, 62]. Ang/Tie axis is another signaling pathway that significantly associated with angiogenesis. Ang-1 participates in migration, adhesion, survival, and proliferation of endothelial cells, whereas the role of Ang-2 remains controversial depending on the cytokine microenvironment. Ang-2 competes with Ang-1 to bind the receptor and leads to apoptosis of endothelial cells and disruption of vasculature integrity, meanwhile contributes to angiogenesis by binding VEGF [63]. The controversial effects of Ang-2 in angiogenesis warrant intensive study in the future.

Another study taken by Passaro et al. [64] indicated that AML microenvironment not only altered vascular density, but also enhanced vascular permeability. Using intravital two-photon microscopy to analyze vascular permeability in human AML engrafted mice with various sizes of TRITC-dextran acted as tracer, they found a significant leakage of the dextran outside the vasculature in transplanted mice compared with controls, with AML engrafted area becoming the leakiest area. Enhanced vascular permeability characterized with increased leakiness impairs drug delivery and reshapes microenvironment into chemo-resistant sanctuary. Therefore, restoring the damaged vascular permeability could serve as an adjuvant treatment strategy in combination with traditional chemotherapy treatment.

### BMAT remodeling

Remodeling of bone marrow adipocytes is intimately involved in AML development. Due to the uncontrolled expansion of leukemic cells in the limited bone marrow cavity, the living space of adipocytes is squeezed, inducing a series of adipocyte remodeling process like morphological changes and lipolysis [65].

Previous evidence revealed that BMAT has a controversial effect on normal hematopoiesis. While in the leukemia environment, existing evidence unanimously supported the favorable role of the transformed BMAT in promoting survival and proliferation of leukemic cells. A study examined the adipocyte-leukemic cell interactions and elucidated that BMAT was modulated into a lipolytic state under the impact of AML cells, with subsequent releasing free fatty acids to maintain nutrient supply for leukemic cells within exuberant metabolism [66]. Later in a study exploring the mechanisms behind the remodeling of bone marrow adipocyte, Lu et al. [67] reported that growth differentiation factor-15 (GDF15), a secreted propeptide highly expressed in leukemic cells, could promote the morphological transition of small adipocytes from larger adipocytes when released to the bone marrow cavity. they proceeded to elucidate that the down-regulation of TRPV4 (a calcium channel on the adipocyte membrane) mediated by GDF15 played an important role in the morphological transition process [68]. Previous study found that highly expressed lipolytic gene was expressed in small adipocytes in mice [69]. In the study of Lu et al. [67], the lipolytic activity of GDF15-induced small adipocytes apparently enhanced with the elevated expression of lipolytic genes including *HSL* and *ATGL*. In turn, the large amounts of free fatty acids produced by lipolysis function satisfied energy requirements for leukemic cells to support survival of leukemic cells [66].

Besides providing energy for leukemic cells, the disruption of adipocyte bone marrow niche also impaired endogenous myelo-erythropoiesis [22]. Proliferation of normal BMAT induced by PPARγ agonists could rescue healthy hematopoiesis and repress leukemia development, indicating that targeting BMAT could be regarded as a potential strategy to arrest leukemia progression.

### Sympathetic nervous system alteration

Studies have found that sympathetic neuropathy accelerated the progression of AML. Hanoun et al. [70] revealed that in an MLL-AF9 AML murine model, bone marrow infiltration of malignant cells manipulated neuropathy of sympathetic nervous system (SNS) and reinforced AML progression. This process disrupts nestin GFP^+^ cell quiescence, leading to osteoblastic differentiation of the bone marrow MSCs at the expense of the reserved space for bone marrow cavity. The manipulated microenvironment with SNS denervation facilitates expansion of leukemic cells and finally becomes not suitable for normal HSCs. Additionally, blockade of β2-adrenergic tone in these mice was found associated with extended leukemic cells proliferation and poor outcome, while the administration of an β2-adrenergic agonist led to reduction of LSCs and prolonged survival, indicating that signal alteration of β2-adrenergic associated pathway might be involved in leukemia progression. In another study, treated with β3-adrenergic stimulators, the restoration of sympathetic regulation on nestin^+^ MSCs alleviated myeloproliferative neoplasms progression [71]. In general, these studies have suggested that normal SNS maintenance contributed to preservation of healthy HSCs and limitation of LSC expansion, prompting a future direction for ameliorating the malignant microenvironment.

### Cytokines and adhesion molecules

The malignant BMM mediates the anchorage of LSCs by up-regulation of adhesion factors and chemokines, thus activating pro-survival pathways and providing a stable shelter for LSCs. Like normal HSCs, the majority of LSCs also express CXCR4 on their surface and migrate in response to CXCL12. Although AML cells exhibiting varying degrees of differentiation expressed different CXCR4 levels, Tavor et al. [72] showed that internal CXCR4 expression was up-regulated in all AML cases, including cells which do not express surface CXCR4. Indeed, the CXCL12/CXCR4 axis has been illustrated played crucial role in LSCs survivability and proliferation. Previous studies showed that the activated binding of CXCL12 to CXCR4 activated downstream pro-survival and proliferative pathways including the JAK/STAT, PI3K/AKT, and MEK/ERK pathways [73, 74]. Correspondingly, inhibition of CXCL12/CXCR4 axis significantly impaired AML cell proliferation In vitro [75]. CXCR4 has also been shown to play a key role in LSCs migration and homing. Burger et al. [76] observed that CXCR4 activation was associated with migration of CD34^+^ HSPCs and AML cells beneath marrow stromal cells. Voermans et al. [77] investigated the phenotype of the migrated cells and showed that significantly higher migration was observed in LSCs compared to other LSCs-derived cells in AML. The prognostic value of CXCL12/CXCR4 axis in leukemia has been studied to push forward risk-stratified therapeutic strategies. It was reported that elevated CXCR4 expression in LSCs defined a high relapse rate and a significantly poor outcome [78]. Taken together, these evidences revealed up-regulating of CXCL12/CXCR4 axis contributed to AML development by promoting leukemia cell proliferation and homing capacity.

Adhesion to the niche is a fundamental step to AML pathogenesis and progression. In AML environment, Jacamo et al. [79] have shown that very late antigen-4 (VLA-4) was highly expressed in AML cells. The interaction of VLA-4 with vascular cell adhesion molecule-1 (VCAM-1) on stromal cells targeted pro-survival and proliferative pathways in both AML cells and stromal cells through NF-κB pathway, ultimately committing to chemotherapy resistance. Previous studies on the role of VLA-4 in AML prognostic values have reported controversial findings. A study on 36 AML patients demonstrated that expanded VLA-4 expression predicted an unfavored prognosis [80]. Surprisingly, several independent studies for pediatric and adult patients have shown that an elevated expression of VLA-4 was linked with a better prognosis [81, 82]. Thus, well-designed studies with large-sample size are required to prudently certify the connection between elevated VLA-4 expression and outcome in AML. Several studies reported that an enhanced E-selectin level and an elevated binding capacity to LSCs were found in murine models of AML [83, 84]. A study revealed that the adhesion of E-selectin in AML led to the activation of the wnt pathway and hedgehog pathway of AML blasts to facilitate the survivability of AML blasts in the microenvironment [85]. Jin et al. [86] revealed that leukemic repopulation was markedly decreased through administration of CD44 antibody in human AML transplanted mice. Another study showed that the expression of CD44 variant exons in AML cells is more common and complex compared to normal hematopoietic cells [87]. Understanding how these adhesion molecules interplay with each other to affect leukemogenesis contributes to the developing of innovative therapeutic interventions.

### Hypoxia and levels of ROS

It has been reported that while no detectable difference of hypoxic level has been observed between healthy bone marrow and leukemic bone marrow, leukemic cells could better adapt in hypoxic microenvironment compared to HSCs. Thus, the hypoxic microenvironment efficiently permit an expansion of leukemic subgroup in AML progression [88]. Several studies have investigated the role of activated HIF-1α and HIF-2α in AML progression. Nonetheless, there remain disputes with the effect of HIFs, with HIFs being regarded as either oncogenes or tumor suppressor genes in AML. *HIF-1* or *HIF-2* knockdown in AML patient samples compromised their ability to reconstitute AML upon transplantation into recipient mice [53, 89]. Abdul-Aziz et al. demonstrated that hypoxia in bone marrow induced high levels of macrophage migration inhibitor factor (MIF) in AML cells to promote survivability and proliferation of AML cells in vivo [90]. Another study found that co-expression of *RUNX1* and *ASXL1* mutations enhanced transcription of *HIF-1α* and its target gene *inhibitor of DNA binding 1 *(*ID1*) expression [91]. Through initiating activation of AKT signaling pathway, *ID1* played a key role in excessive proliferation promotion of leukemic cells [92]. Intriguingly, evidence revealed that HIF-1α also exerted an opposite effect of anti–AML progression by inducing AML cells to undergo differentiation. A study showed that by regulating the miRNA network and activating downstream p21 and STAT3, HIF-1α inhibited proliferation and induced differentiation of AML cells [93]. Moreover, a research using conditional *Hif-1α* knockout murine model reported that any improvement of the outcome from *Hif-1α* deletion failed to be observed. In contrast, a faster development of AML and an enhanced aggressive phenotype were observed in this murine model with deletion of *Hif-1α* [94]. Similar outcomes were found in HIF-2α. Vukovic et al. [95] demonstrated that although HIF-2α obstructed LSCs development, it had no suppressive effects on LSCs maintenance in a conditional genetic model. These studies have challenged the general notion of potential strategies on repressing HIF signaling pathway. The possible explain for the controversy could be the different genetic models (mouse versus human) the investigations developed. Besides, even in the same individual, the effect of hypoxia on quiescent LSCs and relatively mature leukemic cells could be quite different. Moreover, there may exist unknown hypoxia-independent pathway downstream of HIF to compensate HIF inhibition. Therefore, the research results of HIF inhibition in single study cannot be generalized and future work should pay attention to resolve these questions in heterogeneous genetic subtypes of AML.

The generation of endogenous ROS is limited by the hypoxic BMM [96]. ROS remains heterogeneous within most LSCs in a reduced state (ROS-low) to maintain LSCs dominance and a relatively long-term survival. Intriguingly, although LSCs are consistent with HSCs in the oxidation state, the energy generation strategies of LSCs are significantly different. HSCs can effectively achieve energy homeostasis by glycolysis, while LSCs are highly dependent on oxidative respiration instead of glycolysis to remain activity for survival. Additionally, a preferential elevation of BCL-2 was found in LSCs with low ROS compared to those with high ROS, suggesting the potential participation of anti-apoptotic protein in remaining mitochondrial activity to avoid accumulation of ROS [97]. Moreover, attention should be paid on identifying the specific self-renewal signaling pathway in LSCs but not shared by normal HSCs. Zhang et al. [98] revealed that Slug/Slc13a3 signaling pathway enhanced intracellular ROS level to promote the initiation and maintenance of LSCs. Accordingly, the differential distribution of ROS level among individuals, in space, and across time requires in-depth mechanism research.

## Role of interaction between BMM and LSCs in environment-mediated drug resistance (EMDR)

EMDR is one form of extrinsic primary drug resistance. It happens when the surrounding microenvironment temporarily protect tumor cells from apoptosis, thus leading to the preservation and even expansion of adaptive tumor cell subsets [99]. Based on different resistance mechanisms mediated in the tumor microenvironment, EMDR can be divided into two categories: soluble factor-mediated drug resistance (SFM-DR) and cell adhesion-mediated drug resistance (CAM-DR). SFM-DR is induced by soluble molecules secreted by tumor stroma, which including CXCL12, VEGF, IL-6, G-CSF and other soluble molecules. CAM-DR is a direct cell-to-cell contact mediated by adhesion factors like selectins, integrins, cadherins and components of ECM, such as fibronectin, laminin, OPN and collagen.

Meads et al. [100] proposed that the development of drug resistance could be divided into three major stages: (1) tumor cells homing to BMM; (2) maintaining in the bone marrow protective shelter mediated by EMDR; (3) evolving an acquired drug resistance phenotype. The CXCL12/CXCR4 interaction, taking charge of homing and maintaining hematopoietic cells in the bone marrow, frequently presents in the first stage. A study reported that CXCR4 blockade augmented the sensitivity of AML cells to apoptosis induced by the FMS-like tyrosine kinase-3 gene (*FLT3*) inhibitor sorafenib in stromal cocultures [73]. Previous studies have shown that activation of the STAT5/PIM kinase axis tightly participated in leukemogenesis accompanied with *FLT3*-ITD. Later research confirmed the critical role of PIM-1 activity for high levels of surface CXCR4 expression to regulate homing and migration of hematopoietic cells and LSCs [101]. Additionally, CXCL12 activates VLA-4-dependent migration, which contributes significantly to CAM-DR phenotype [102]. SFM-DR and CAM-DR play essential roles in the second stage of chemo-resistance development. IL-6, an important cytokine mediating proliferation and differentiation of HSCs, is primarily secreted by bone marrow stromal cells and various hematological malignancy cells [103]. Aberrant activation of STAT3 has been found to induce anti-apoptosis by up-regulation of BCL-2 family in a wide range of malignances including AML [104]. Later studies in pediatric AML patients demonstrated that elevated IL-6 induced chemo-resistance and exacerbated disease progression through enhanced STAT3 activity, which was associated with a poor outcome [105]. In addition to soluble factors, LSCs dormancy is also related with adhesion factors represented by integrin family. Matsunaga et al. [106] indicated that the interaction between VLA-4 on leukemic cells and fibronectin on stromal cells activated the PI3K/AKT/BCL-2 signaling pathway to acquire drug-induced apoptosis resistance. Another study using antibodies targeting VLA-4 observed that blockade of VLA-4 in combination with CD3 redirection sensitized cytotoxicity [107]. Recently, a study investigated the role of integrin α7 in AML cells and found that the elevated expression of integrin α7 in AML activated ERK signal and impaired sensitivity to the stromal-induced drug resistance [108, 109]. The selective pressure of cumulative cytotoxic therapies over time gradually contributes to the transformation from random genetic mutations to acquired resistance in these surviving cell subsets, eventually causing residual leukemic cell expansion and disease relapse. EMDR served as protective resistance via alteration of post-transcription, until the ultimate emergence of acquired resistance phenotype with common alterations of transcription levels evolving in leukemic cells [110]. This transformation process of resistance suggests that innovative therapeutic strategies toward the microenvironment should be applicated from the onset of initial relapse, instead of not being considered until disease develops into an advanced stage.

Hypoxia-induced HIF expression primarily favors the quiescence of AML cells [111]. Considering that the current mainstream chemotherapy regimens represented by combination of cytarabine and daunorubicin are aimed at circulating cells, quiescence remarkably enhanced the chemotherapy resistance of AML cells. Besides, hypoxia durably activates the PI3K/AKT/mTOR signaling pathway, inducing anti-apoptosis and weakening the chemotherapy sensitivity of AML cells [112]. A recent study reported that hypoxia enhanced the expression of receptor tyrosine kinase (RTK) AXL to trigger an enhanced AXL mediated drug resistance to quizardinib in *FLT3*-ITD transplanted mice [113]. Another research revealed that HIF-dependent upregulation of BMX kinase reduced FLT3 inhibitor sorafenib sensitivity through compensatory pro-survival signaling pathway in a murine *Flt3*-ITD model [114]. Recently, Sauvageau et al. [115] revealed the metabolic differences between chemotherapy-resistant AML cell subgroups and chemotherapy-sensitive AML subgroups. They found that chemotherapy-sensitive AML cell subgroups did not require mitochondrial respiration to produce energy, while the chemotherapy-resistant AML cell subgroups highly depended on NADH dehydrogenase activity for survival. The Warburg effect, considered as a key feature of cancer, describes that tumor cells produce energy by glycolysis without oxygen. The discovery in AML drug-resistant cells overturned universality of this perception, with more attempts in inhibiting mitochondrial metabolism to target chemotherapy-resistant AML cell subgroups being needed.

MSC-EVs were found in a variety of cancers to induce resistance to chemotherapy agents [116]. Recently, several studies have probed into mechanisms of MSC-EVs in AML resistance. Lyu et al. [117] reported that exosome-treatment of AML cells acquired enhanced resistance to cytarabine by up-regulating S100A4, an important effector participated in cell adherence and cytokine regulation. Another study revealed that after exosomes encapsulating fibroblast growth factor 2 (FGF2) in bone marrow stromal cells, they were subsequently endocytosed by leukemic cells and protected leukemic cells from tyrosine kinase inhibitors (TKIs) [118]. These evidences suggested that inhibiting the synthesis and release of exosomes could be a potential strategy to overcome drug resistance. In addition, emerging studies have supported that the characteristics of high-efficient loading of exosomes can be considered for drug delivery, with its natural transmembrane capacity as advantage [116].

Mitochondrial transfer in AML describes a novel cell-to-cell communication mechanism through delivery of functional mitochondria from donor stromal cells to recipient leukemic cells [119]. An increase in mitochondrial mass enabled an elevated rate of oxidative phosphorylation and an obvious AML cell survival advantage against cytotoxic chemotherapy in the recipient leukemic cells co-cultured with bone marrow stromal cells [120]. The xenograft model in immunodeficiency mice further confirmed the occurrence of mitochondrial transfer in AML and its effect against chemotherapy-induced apoptosis. Marlein et al. [121] described that NADPH oxidase-2 (NOX2) activation led to increasing oxidative stress, stimulating mitochondrial transfer from stromal cells to AML cells through AML-derived tunneling nanotubes which acted as bridge. Moreover, Blocking NOX2 inhibited mitochondrial transfer, increased AML apoptosis, and improved AML mouse survival. Remarkably, any significant effect on normal CD34+ cell survival failed to be detected, suggesting that targeting NOX2-driven mitochondrial transfer appears to be a novel therapeutic strategy in AML. Later Marlein et al. [122] focused on stromal cells with loss of mitochondria after mitochondrial transfer, discovering that the up-regulation of peroxisome proliferator-activated receptor gamma coactivator 1-alpha (PGC-1α) led to an increased accumulation of functional mitochondria in stromal cells. In this way, stromal cells restore its metabolic capacity and keep a long-term balance of metabolism.

## Strategies targeting BMM in AML

**Fig. 2:**
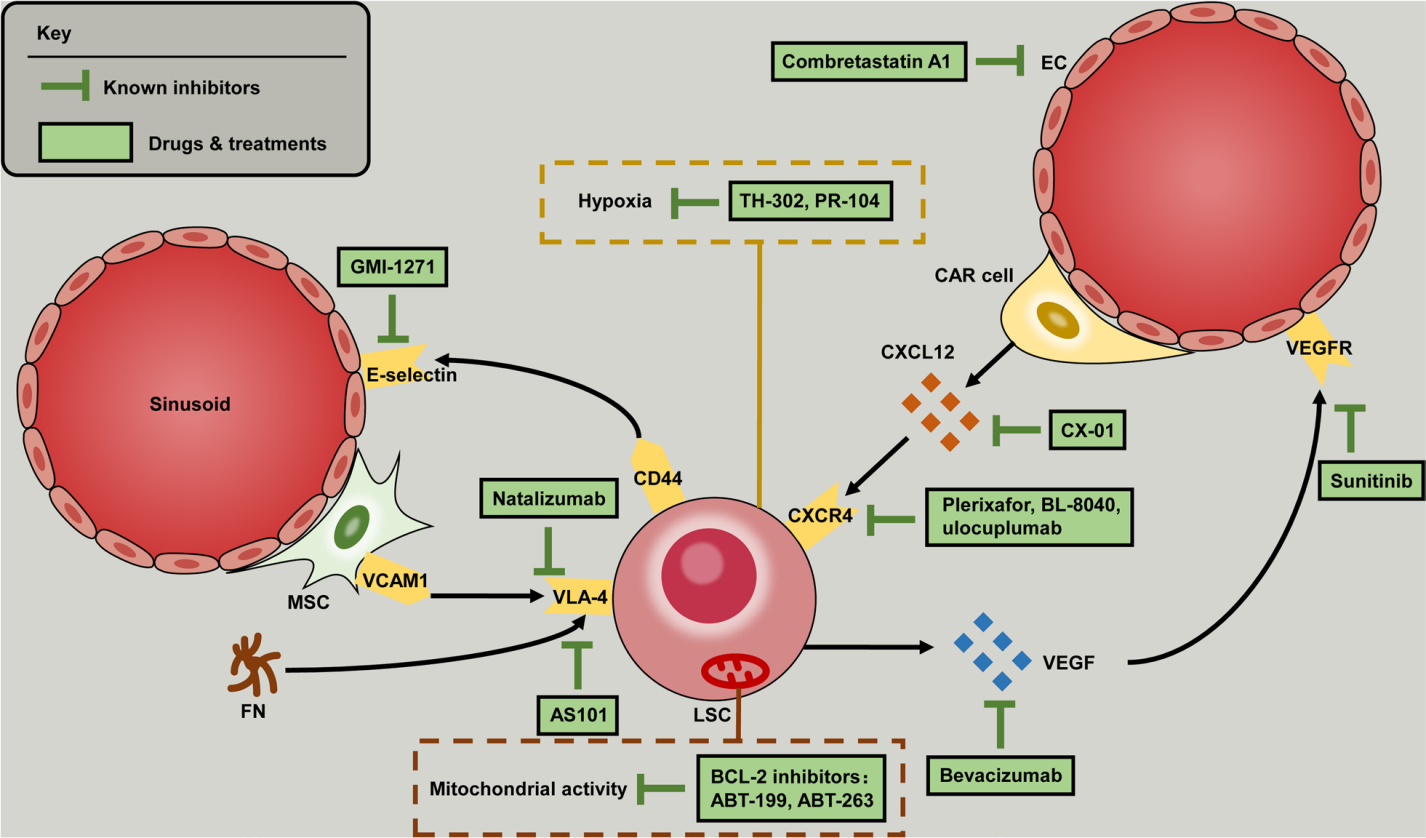
Strategies to target the bone marrow microenvironment in AML. *AML* acute myeloid leukemia, *EC* endothelial cell, *CXCL12* C-X-C motif chemokine 12, *CXCR4* C-X-C chemokine receptor 4, *VLA-4* very late antigen 4, *VCAM-1* vascular cell adhesion molecule 1, *VEGF* vascular endothelial growth factor, *VEGFR* vascular endothelial growth factor receptor, *LSC* leukemic stem cell, *CAR cell* CXCL12-abundant reticular cell, *FN* fibronectin, *BCL-2* B-cell lymphoma-2

As previously mentioned, extensive evidence indicates that the alteration of BMM is intimately associated with AML development and therapeutic resistance. Several prominent BMM changes including remodeling of the vasculature, alteration of cytokine levels as well as adhesion capacity, adaptability to hypoxic microenvironment, and approach to maintain a low cellular oxidative in LSCs. Several classes of therapeutic agents have been designed to effectively target these pathological processes of environmental alterations. Currently, combined therapies targeting LSCs mobilization from protective shelters have been tested under various phases of clinical trials. In addition, cell-mediated drug-delivery system facilitating the targeted transport of chemotherapeutic agents provides a new insight to improve the efficacy of current chemotherapy regimens, although it has not been confirmed by clinical trials [123]. Here we summarize current available strategies targeting BMM that might represent potential directions for future adjunctive therapeutics (Table 1, Fig. 2).

**Table 1:** Summary of clinical trials targeting the bone marrow microenvironment in AML

Target	Regimen	ClinicalTrial.gov Identifier	Patient population	Phase	Response
VEGF	Bevacizumab + cytarabine/idarubicin	NCT 00096148	Untreated, < 60 years	II	
	Bevacizumab + cytarabine/mitoxantrone hydrochloride	NCT00015951	Relapsed/refractory, ≥ 18 years	II	CR 33%
RTK	Sunitinib	NCT 00783653	Untreated, FLT3-ITD, ≥ 60 years	I/II	CR + CRi 59%
Tubulin	Combretastatin A1 + cytarabine	NCT02576301	Relapsed/refractory, ≥ 18 years	I/II	CR + CRi 15%
CXCR4	Plerixafor + decitabine	NCT 01352650	Untreated, ≥ 60 years	I	
	Plerixafor + cytarabine/daunorubicin	NCT 00990054	Untreated, 18–70 years	I	CR 67%
	Plerixafor + sorafenib/G-CSF	NCT 00943943	Relapsed/refractory, FLT3-ITD, ≥ 18 years	I	CR + CRi 36%
	Plerixafor + mitoxantrone/etoposide/cytarabine (MEC)	NCT 00512252	Relapsed/refractory, 18–70 years	I/II	CR + CRi 46%
	Plerixafor + G-CSF/mitoxantrone/etoposide/cytarabine (MEC)	NCT 00906945	Relapsed/refractory, 18–70 years	I/II	CR + CRi 30%
	Plerixafor + cytarabine/etoposide	NCT 01319864	Relapsed/refractory, 3–29 years	I/II	
	Plerixafor + G-CSF/ busulfan/fludarabine/thymoglobulin	NCT 00822770	Allo-SCT, 18–65 years	I/II	
	Plerixafor + daunorubicin/clofarabine or daunorubicin/cytarabine	NCT 01236144	Untreated, ≥ 60 years	I/II	
	Plerixafor + clofarabine	NCT 01160354	Untreated, ≥ 60 years	I/II	
	Plerixafor + fludarabine/idarubicin/cytarabine/G-CSF (FLAG)	NCT 01435343	Relapsed/refractory, 18–65 years	I/II	
	BL-8040 + cytarabine	NCT 01838395	Relapsed/refractory, 18–75 years	II	CR + CRi 39%
	BL-8040 + atezolizumab	NCT 03154827	Relapsed/refractory, ≥ 60 years	Ib/II	
	Ulocuplumab	NCT 01120457	Relapsed/refractory, ≥ 18 years	I	CR + CRi 51%
CXCL12	CX-01 + cytarabine/idarubicin	NCT 02056782	Untreated, ≥ 60 years	II	CR 92%
	CX-01 + cytarabine/idarubicin	NCT 02873338	Untreated, ≥ 60 years	II	CR + CRi 89%
	CX-01 + azacytidine	NCT 02995655	Relapsed/refractory, ≥ 18 years	I	
E-Selectin	GMI-1271 + idarubicin/mitoxantrone/etoposide/cytarabine (MEC)	NCT 02306291	Relapsed/refractory, or untreated, ≥ 60 years	I/II	
	GMI-1271 + mitoxantrone/etoposide/cytarabine (MEC) or fludarabine/cytarabine/idarubicin (FAI)	NCT 03616470	Relapsed/refractory, 18–75 years	III	
	GMI-1271 + daunorubicin/cytarabine	NCT 03701308	Untreated, ≥ 60 years	II/III	
VLA-4	AS101 + chemotherapy	NCT 01010373	Untreated, ≥ 60 years	II	
Hypoxia	TH-302	NCT 01149915	Relapsed/refractory, ≥ 18 years	I	CR + CRi 5%
	PR-104	NCT 01037556	Relapsed/refractory, ≥ 18 years	I/II	CR + CRp 32%

*AML* acute myeloid leukemia, *VEGF* vascular endothelial growth factor, *RTK* receptor tyrosine kinase, *CR* complete remission, *CRi* complete remission with incomplete count recovery, *CRp* complete remission with incomplete platelet count recovery, *G-CSF* granulocyte-colony stimulating factor, *Allo-SCT* allogeneic stem cell transplantation

### Strategies from anti-angiogenesis to guidance of early vasculature normalization

Several anti-angiogenic strategies targeting angiogenic factor signaling pathways have been attempted before. Unfortunately, up to date, most clinical trials on AML with agents targeting angiogenesis have shown disappointing results. VEGF is one of the most critical factors for angiogenesis. Bevacizumab is a widely investigated humanized recombinant monoclonal antibody targeting VEGF which has been applied in treatment for solid tumors. An early phase II clinical trial designed a regimen with standard induction chemotherapy followed by bevacizumab and reported a favorable CR, suggesting the potential clinical activity of bevacizumab (NCT00015951) [124]. While another study reported that although significantly decreased VEGF level was detected in patients with relapsed/refractory AML treated with bevacizumab, no or only modest anti-leukemic efficacy was observed [125]. Later, a randomized phase II trial of bevacizumab did not show any improvement in the therapeutic outcome of elderly AML patients, either (NTR904) [126]. Clinical trials on other VEGF inhibitors like lenalidomide and thalidomide displayed neither limited nor controversial efficacy in patients with relapsed/refractory AML [127–129]. VEGF mediates downstream effects through VEGFR-2, a receptor which exhibits tyrosine-kinase activity. TKIs are a family of small molecules that aim at diverse categories of targets with tyrosine kinase activity such as VEGF signals through blocking downstream RTK. In a phase I/II study (NCT00783653), favorable CR rates and long-term outcome have been observed in patients treated with combined therapies of TKI sunitinib and standard chemotherapy [130]. The activation of other pro-angiogenic growth factors such as Ang/Tie-2 can also stimulate angiogenesis, thereby contributing to the major drug resistance mechanism of VEGF-targeted therapy. New generation of anti-angiogenic targeted drugs blocking both VEGF and Ang/Tie-2 pathway to delay the occurrence of chemotherapy resistance could be the direction of future research.

In addition to restore normal vascular system, vascular disrupting agents (VDAs) to destabilize leukemic blood vessels suggest an alternative strategy. Combretastatin A1 (OXi450) is a novel dual-function agent with both vascular disruption and cytotoxic activity against AML cells. Through binding tubulin, combretastatin A1 mediates microtubule depolymerization and cytoskeleton collapse of endothelial cells, eventually causing obstruction of the tumor vasculature due to enlarged endothelial cells [131]. A study tested combretastatin A1 alone and in combination with bevacizumab in xenotransplant murine models reported that combretastatin A1 treatment alone of human AML chloromas led to vascular disruption in leukemia cores and increased apoptosis. Furthermore, combination with bevacizumab abrogated VEGF-A-rich vascular rims and led to enhanced leukemia regression, suggesting that compared to monotherapy, multi-targeted anti-angiogenesis therapy appears to be a superior approach. In addition, the observed regression of leukemia engraftment couldn’t be completely explained by alteration of blood vessel density. Further research confirmed that combretastatin A1 also exhibited direct cytotoxic effects on leukemic cells, which was mediated by generation of ROS [132]. Later, a phase I clinical trials on a novel combination therapy with combretastatin A1 and cytarabine in patients with relapsed/refractory AML displayed a prolonged OS and well tolerance (NCT02576301) [133].

The efficacy of anti-angiogenic therapies in improving AML patient outcomes remains controversial, Duarte et al. [134] further investigated vascular remodeling of different bone marrow region in AML and found central marrow remaining vascularized while endosteal regions being remodeled and even ultimately degraded, which is associated with loss of normal hematopoiesis. This finding suggested that precisely targeting endosteal vasculature to prevent degradation instead of taking anti-angiogenic agents appears to be a possible alternative approach. It has been reported that blast differentiation together with reversal of pancytopenia was observed in AML treated with iron chelator deferoxamine [135]. Later, Duarte et al. revealed that deferoxamine significantly rescued degradation of the endosteal vasculature and HSCs, as well as supporting homing of HSCs. However, little remission of AML progression in deferoxamine-treated mice was observed. Accordingly, current major challenge for anti-angiogenic strategies remains. Since restoring the damaged vascular permeability could serve as an adjuvant treatment strategy in combination with traditional chemotherapy treatment and clinical strategies with single anti-angiogenic agent targeting AML did not display a notable efficacy, strategies from mono-target to multi-target, from generally anti-angiogenesis to remodeling regional normal vascularity are deserved to be explored.

### Targeting cytokine secretion and adhesion molecules in BMM of AML

The primary purpose of a series of strategies targeting chemokines and adhesion factors is to mobilize the resting LSC from the protective niche. By far the clinical trials based on this steering philosophy have achieved temporary success. CXCR4 inhibitors have been widely investigated as the most prominent candidate. The disruption of CXCR4/CXCL12 interaction blocks downstream signals such as PI3K/AKT and MAPK pathways, mediating AML cells mobilizing from their shelter to augment targeted delivery of chemotherapeutic agents. The first preclinical trial focusing on blocking CXCL12/CXCR4 axis used the CXCR4 inhibitor AMD3465. By blocking CXCR4, AMD3465 led to suppression of stroma-activated PI3K/AKT and MAPK pro-survival pathways in *FLt3*-mutated AML cells. In a murine xenograft model, AMD3465 promoted *FLT3*-mutated AML cells mobilizing into the peripheral blood, eliminated the stroma-mediated support of the microenvironment, and thus enhanced chemo-sensitivity of leukemic cells to cytarabine and FLT3 inhibitor sorafenib [73]. Plerixafor (also known as mozobil, and AMD3100) is a small selective molecular inhibitor of CXCR4 which was first identified as an anti-HIV agent. The combination of plerixafor and G-CSF has been approved by the US Food and Drug Administration (FDA) for mobilization of autologous transplantation in patients with multiple myeloma (MM) and non-Hodgkin’s lymphoma (NHL) since 2008. In a murine model of acute promyelocytic leukemia, addition of plerixafor to cytarabine significantly eased leukemia burden and prolonged OS compared with cytarabine alone [136]. In an initial phase I/II study (NCT00512252), 52 patients with relapsed/refractory AML were treated with a combination of plerixafor and mitoxantrone, etoposide, cytarabine (MEC) regimen. The regimen appeared to be well tolerated and achieved a relatively satisfactory CR and CR with incomplete blood count recovery (CRi) rate of 46%. Moreover, the mobilization rate of AML blasts into peripheral blood was almost doubled [137]. However, another study using the combination of G-CSF and plerixafor in conjunction with MEC regimen showed no improved remission rates (NCT00906945) [138]. Recently, in a phase I study (NCT00943943), 28 relapsed/refractory, *FLT3*-ITD-mutated AML patients were treated with combinatorial sorafenib, G-CSF, and plerixafor regimens. A CR/CRi rate of 36% and a blast mobilization of 58.4 fold were shown in this study [139]. BL-8040 (also known as BKT140) is another selective inhibitor of CXCR4 which could induce a robust mobilization by blocking the binding of CXCL12 to CXCR4 as well as reducing the baseline activity of CXCR4. Besides, BL-8040 also exhibited a direct anti-leukemic effect via inducing apoptosis of AML blasts [140]. In a phase IIa clinical trial with relapsed/refractory AML patients (NCT01838395), combination of BL-8040 with cytarabine evidently triggered the mobilization of blasts into peripheral blood [141]. Ulocuplumab (also known as BMS-936564, and MDX-1338) is a fully human IgG4 monoclonal antibody to CXCR4 with directly pro-apoptotic effects [142]. A CR/CRi rate of 51% was observed in a phase I clinical trial in patients with relapsed/refractory AML treated with ulocuplumab in combination with chemotherapy of MEC (NCT01120457) [143]. CX-01 is a heparin derivative with little or no anticoagulant activity. It blocks CXCL12/CXCR4 axis by binding CXCL12. A randomized phase I clinical trial of untreated AML patients with CX-01 combined with cytarabine and idarubicin (7 + 3) showed an encouraging CR rate of 92% (NCT02056782). In addition, the combination treatment was well tolerated with a rapid hematologic recovery [144]. In consistent with the single-arm pilot study, recently a phase II clinical trial in untreated AML patients with combination of CX-01 and a standard therapeutic regimen resulted in a CR/CRi rate of 89% along with excellent tolerability (NCT02873338) [145].

Adhesion molecules such as VLA-4, CD44, and E-selectin may be regarded as candidate therapeutic targets for tumor migration. Natalizumab (also known as tysabri), a humanized VLA-4 monoclonal antibody which can reduce central nervous system (CNS) lymphocyte infiltration, is in clinical use for the treatment of autoimmune diseases. A durable HSC mobilization has been observed in patients with multiple sclerosis treated with natalizumab [146]. Unfortunately, the clinical application of natalizumab is limited by potential side-effect, JC virus–associated progressive multifocal leukoencephalopathy [147]. Another VLA-4 inhibitor in clinical research is AS101. AS101 results in redox inhibition of VLA-4 by binding fibronectin and suppressing PI3K/AKT/BCL-2 signaling to increase chemo-sensitivity [148]. A murine xenograft AML model showed that AS101 improved chemo-sensitivity of leukemic cells and prolonged survival in mice after chemotherapy. A phase II clinical trial of AS101 is currently underway in untreated elderly patients (NCT01010373). In addition to VLA-4, another important adhesion molecule is E-selectin. GMI-1359 was first reported as a small molecule that simultaneously inhibited both E-selectin and CXCR4, significantly promoted mobilization and resulted in prolonged survival in a *Flt3*-ITD AML murine xenograft model [149]. GMI-1271 (also known as Uproleselan) is another specific small molecule inhibitor of E-selectin. A murine xenograft model showed that Blockade of E-selectin with GMI-1271 enhanced the effect of combination with daunorubicin and cytarabine, significantly improved overall treatment efficacy, and prolonged mice survival [150]. A phase I/II clinical trial on GMI-1271 with a combination of MEC or fludarabine, cytarabine, idarubicin (FAI) is completed and GMI-1271 showed promising prospects in relapsed/refractory AML patients (NCT02306291). Two other clinical trials are currently underway (NCT03616470, and NCT03701308). Furthermore, another small molecule E-selectin inhibitor GMI-1687 is currently undergoing tests in preclinical trials. Finally, CD44 monoclonal antibody H90 showed a significant reduction of the leukemic burden in xenograft AML models. Moreover, a failure of the secondary engraftment was observed using leukemic cells taken out from primary mice treated with H90, suggesting that CD44 directly targeted LSCs [86]. Accordingly, the therapeutic potential of targeting adhesion molecules deserved to be noted.

### Targeting hypoxia and inducing elevated ROS in BMM

Targeting hypoxia is an emerging strategy for the treatment of hematologic malignancies. Considering the characteristics of HIF contributing to LSC maintenance, combination of HIF inhibitors with traditional chemotherapy regimens targeting circulating cells appears to be a potential therapeutic strategy to break the dormancy and efficiently move LSCs out to receive chemotherapy toxicity. Nonetheless, the results in preclinical trials challenge the general notion of LSC sensitization by HIF inhibition, indicating the mechanism and effects of HIF inhibition should be further investigated before being applied in clinic. Indeed, based on the characteristics of hypoxic niche, hypoxia-activated prodrugs (HAPs) have been designed as an alternative strategy to overcome the problem with hypoxia induced resistance. Under hypoxic environment, HAPs release cytotoxic agents which are activated by enzymatic reduction, thus selectively targeting hypoxic malignancies. TH-302 (also known as evofosfamide) is a 2-nitroimidazole-linked prodrug. It is designed to release a DNA-crosslinking mustard alkylating agent bromo-isophosphoramide mustard (Br-IPM) within hypoxic subregions. Portwood et al. [151] demonstrated that within TH-302 treatment, previously chemo-resistant human AML cells under hypoxic conditions exhibited enhanced sensitivity to cytarabine and underwent extensive apoptosis induced by cytarabine. Benito et al. [152] reported that in a murine AML model, TH-302 synergistically with TKI sorafenib induced apoptosis of leukemic cells and prolonged survival compared with treated alone with sorafenib. TH-302 in combination with traditional chemotherapy has been tested in clinical trials of solid tumors like soft tissue sarcoma and pancreatic cancer [153, 154]. In AML, a phase I clinical trial on TH-302 has been completed (NCT01149915), in which a rapid but transient cytoreduction was observed in the majority of refractory AML patients [155]. Moreover, evidence supports that combining TH-302 therapy with other chemotherapeutic agents (anthracyclines and topoisomerase inhibitors) may be an optimization approach [156, 157]. Another HAP PR-104 has been evaluated in treating patients with refractory/relapsed AML in a phase I/II study (NCT01037556), in which an anti-leukemic activity together with primary toxicity myelosuppression was observed [158]. Further investigations are required to determine the therapeutic efficiency and safety of HAPs in AML, as well as combination therapies with traditional anti-leukemia regimens.

Since most resting LSCs with low ROS abnormally overexpress BCL-2, targeting BCL-2 inhibition appears to be strategies to destroy oxidative phosphorylation and accelerate the elimination of these resting LSCs. Lagadinou et al. [97] demonstrated that BCL-2 inhibitor ABT-263 effectively targeted the LSC enriched ROS-low population by impairing their mitochondrial energy generation capacity and redox control. NF-E2-related factor 2 (Nrf2) induces an antioxidant response pathway which leads to an augmented mitochondrial ROS induction. A preclinical study demonstrated that after repressing the inhibition of Nrf2, an enhanced level of ROS and apoptosis was found in combined treatment hypomethylating agents and venetoclax (ABT-199) treated on AML compared to treatment of hypomethylating agents alone [159]. Nevertheless, additional studies on BCL-2 inhibitors are required to confirm the effect of apoptotic induction through elimination of oxidative phosphorylation.

## Conclusions and outlook

In summary, although significant progress has been made in the treatment of AML, a dismal prognosis remains in the majority of AML patients. Due to the highly heterogeneous character of AML, existing chemotherapy therapies and targeted therapies aimed to leukemic cells can hardly get rid of all subclones of LSCs, resulting in the occurrence of minimal residual disease (MRD) and high rate of relapse.

With development in transgenic animal models and imaging technologies, numerous observations indicated that LSCs could remodel bone marrow niche and the educated-BMM provided a protective shelter to promote chemotherapy resistance. Since complex interactions with the BMM influence survivability and progression of LSCs, focusing on the bidirectional interplay appears to be an attractive strategy to identify new druggable targets and improve the outcome of AML. In our view, by far targeting chemokines and adhesion factors represented by CXCL12/CXCR4 axis and E-selectin to mobilize leukemic cells from their protective niche tend to be one of the most successful strategies for targeting resting leukemia cells, with emerging clinical trials demonstrating the effectiveness of this strategy in conjunction with circulating cell-targeted traditional chemotherapy regimens. In addition to the cell-killing effect of the drug itself, how to achieve high-efficiency drug delivery which hold the capacity to accurately target the BMM has always been a problem. The design of prodrugs targeting the hypoxic domain has undoubtedly opened new possibilities and directions in leukemia drug delivery, with clinical investigations of HAPs currently ongoing for expanded supporting evidence.

Nonetheless, limitations and challenges still exist on the way to future clinical applications. Differences exist in immunophenotypic strategies of HSCs and stromal stem cell subgroups across studies. The lack of reliable markers hinders further studies on the bidirectional interplay between HSCs and BMM. Besides, model of oxygen gradient in BMM needed to be confirmed and specified to quest the relationship between different oxygen gradient and supported-HSCs/LSCs behaviors (maintaining quiescent or being activated) in various niche. What’s more, the disputed role of HIF has not been fully elucidated in the hypoxic BMM and AML development, which required further studies to investigate the impact of HIF on AML cell subsets. In addition, it should be noted that most of in vivo data on phenotypes and mechanisms are obtained through murine models. Due to the limitations of imaging technology, most of studies on the internals of BMM are based on transplanted mice receiving irradiation, instead of dynamic live imaging, with severe changes taking place in BMM which cannot reflect native hematopoiesis [18, 160]. Moreover, considering the existing biological discrepancies in BMM between human and murine models, the transform from mouse models to human populations is limited and need to be performed with caution. Of note, expanding the subclone targets to promote enhanced migration of LSCs out of protective niche may serve as a priming approach adjunctive to cytotoxic chemotherapy to eradicate LSCs. But simultaneously, we must pay attention to explore rational combinational therapies and avoid potential toxicities to remain normal HSCs. Given all of these challenges, a comprehensive and in-depth understanding of the protective shelter BMM is required, in order to prevail the final challenge, elimination of LSCs.

## Data Availability

Not applicable.

## Abbreviations

OS: Overall survival
AML: Acute myeloid leukemia
BMM: Bone marrow microenvironment
LSCs: Leukemia stem cells
HSCs: Hematopoietic stem cells
MSCs: Mesenchymal stem cells
CXCL12: C-X-C motif chemokine ligand 12
ECM: Extracellular matrix
LepR: Leptin receptor
MSC-Evs: Mesenchymal stem cell-derived extracellular vehicles
BMAT: Bone marrow adipose tissue
SCF: Stem cell factor
G-CSF: Granulocyte colony-stimulating factor
HSPCs: Hematopoietic stem/progenitor cells
CNS: Central nervous system
TGF-β: Transforming growth factor β
OPN: Osteopontin
CAR: CXCL12-abundant reticular
CXCR4: C-X-C chemokine receptor 4
VCAM-1: Vascular cell adhesion molecule-1
VLA-4: Very late antigen-4
OCN: Osteocalcin
HIF: Hypoxia-inducible factor
MVD: Bone marrow microvessel density
VEGF: Vascular endothelial growth factor
CR: Complete remission
SNS: Sympathetic nervous system
MIF: Macrophage migration inhibitor factor
ID1: Inhibitor of DNA binding 1
EMDR: Environment-mediated drug resistance
SFM-DR: Soluble factor-mediated drug resistance
CAM-DR: Cell adhesion-mediated drug resistance
FLT3: FMS-like tyrosine kinase-3 gene
RTK: Receptor tyrosine kinase
TKIs: Tyrosine kinase inhibitors
VDAs: Vascular disrupting agents
ROS: Reactive oxygen species
FDA: Food and Drug Administration
MM: Multiple myeloma
NHL: Non-Hodgkin’s lymphoma
MEC: Mitoxantrone, etoposide, cytarabine
FAI: Fludarabine, cytarabine, idarubicin
HAPs: Hypoxia-activated prodrugs
Br-IPM: Bromo-isophosphoramide mustard
MRD: Minimal residual disease
NOX2: NADPH oxidase-2
PGC-1α: Proliferator-activated receptor gamma coactivator 1-alpha
FOXO: Forkhead box O
GDF15: Growth differentiation factor-15
FGF2: Fibroblast growth factor 2
